# Mortality Risk Factors in Appalachian Burn Patients: A 13-Year Retrospective Study

**DOI:** 10.7759/cureus.69658

**Published:** 2024-09-18

**Authors:** Armein Rahimpour, Nathan Fox, Grant Kahley, Gerard V Giangrosso, Karim Abdelgaber, Paul Bown, David A Denning, Curtis Harrison, Barry Rahman

**Affiliations:** 1 General Surgery, Marshall University Joan C. Edwards School of Medicine, Huntington, USA; 2 Plastic and Reconstructive Surgery, Marshall University Joan C. Edwards School of Medicine, Huntington, USA

**Keywords:** appalachian region, burn center, burn injury, mortality risk factors in burn, resource allocation

## Abstract

Introduction

Burn injuries pose a significant public health challenge globally, with Appalachia facing unique obstacles due to its rugged terrain, economic disparities, and limited access to healthcare. Understanding mortality risk factors specific to Appalachian burn patients is crucial for optimizing treatment approaches in this underserved population.

Materials and methods

A retrospective analysis of burn patient data from Cabell Huntington Hospital’s burn intensive care unit (BICU) over 13 years was conducted. Patient records were reviewed, and demographic and clinical variables were analyzed using descriptive statistics and logistic regression models.

Results

Among 1,104 Appalachian burn patients treated at Cabell Huntington Hospital’s BICU between January 2010 and June 2023, advanced age, larger total body surface area (TBSA) burned, inhalation injuries, chronic obstructive pulmonary disease (COPD), and third-degree burns were significant predictors of mortality. Advanced age (p < 0.001, OR: 1.07), larger TBSA burned (p < 0.001, OR: 1.1), inhalation injuries (p < 0.001, OR: 8.34), COPD (p < 0.001, OR: 2.64), and third-degree burns (p < 0.001, OR: 6.45) were significant predictors of mortality. Gender, smoking history, diabetes mellitus (DM), and body mass index did not significantly differ between survivors and deceased patients.

Discussion/conclusion

Our findings underscore the importance of tailored interventions for Appalachian burn patients. Advanced age, pre-existing comorbidities, and burn severity significantly impact mortality risk, emphasizing the need for comprehensive care strategies. Specialized burn centers play a critical role in managing complex burn injuries in underserved regions. Addressing mortality risk factors identified in this study is essential for optimizing burn care outcomes in Appalachia. Tailored interventions and collaborative efforts are needed to improve survival rates and promote health equity for burn patients in underserved regions. Future research should explore additional factors influencing burn outcomes and assess disparities in access to specialized care services.

## Introduction

Despite advances in medical care and technology, burn injuries are still a significant public health challenge globally [1-3]. According to the World Health Organization (WHO), burns rank as the fourth most common cause of traumatic injury worldwide and are associated with significant morbidity and mortality [4]. Although mortality rates have decreased with advances in acute care, severe burns continue to pose formidable clinical challenges, especially in vulnerable populations [3,4].

Appalachian regions in the United States, characterized by rugged terrain, economic disparities, and limited access to healthcare, face unique challenges in managing burn injuries [5]. This population, disproportionately affected by cardiovascular disease, cancer, and other morbidities, often faces unique healthcare challenges and exhibits distinct health behaviors [5,6]. Rural patients generally have decreased access to quality care, including fewer providers, increased travel requirements, and financial burdens, which can negatively impact patient satisfaction [5,6]. West Virginia, made up of a mainly rural population and nestled within the Appalachian Mountains, faces these challenges, with its predominantly rural population lacking access to specialized burn care facilities [5,6]. Residents of this rural region report substandard diet and physical activity behaviors [7]. In turn, it is not shocking that they have the worst health outcomes in the nation [8]. Cabell Huntington Hospital, located in Huntington, WV, serves as the sole provider of burn intensive care unit (BICU) services for the Appalachian region, underscoring the critical importance of understanding the factors influencing burn mortality in this population. This BICU serves the whole state with only six beds.

Many researchers have studied mortality risk factors associated with burn patients worldwide. Multiple different burn mortality predictors and risk factors have been identified. They have highlighted the multifactorial nature of burn injuries and the significance of patient-specific characteristics, injury mechanisms, and pre-existing comorbidities [9-11]. Age, total body surface area (TBSA) burned, inhalation injuries, and pre-existing medical conditions such as chronic obstructive pulmonary disease (COPD) have consistently emerged as predictors of mortality in burn patients [9-11]. However, the applicability of these findings to Appalachian burn patients remains understudied.

This paper aims to address this gap by conducting a comprehensive retrospective analysis of burn patient data from Cabell Huntington Hospital’s BICU over a 13-year period, spanning from January 2010 to June 2023. By focusing specifically on Appalachian burn patients, this study seeks to identify unique mortality risk factors within this population, providing valuable insights for optimizing treatment approaches and resource allocation strategies in a region where specialized burn care resources are limited.

## Materials and methods

The study received approval from the Marshall University Institutional Review Board (IRB no. 2063568-1). Patient records were retrospectively reviewed from our registry at Cabell Huntington Hospital’s BICU. This hospital is an academic teaching hospital, regional referral center, and American College of Surgeons verified Level-2 Trauma Centers in Huntington, WV.

The analyzed medical files belonged to patients who presented between January 1, 2010, and June 1, 2023. Data was obtained by contacting the Information Technology (IT) department. The request included any patient who presented to the hospital with burns. Additionally, we requested age, gender, Injury Severity Score (ISS), and hospital length of stay from the IT team. The initial sample consisted of more than 1,300 patients. All collected data was centralized using Microsoft Excel software (Microsoft Corporation, Redmond, WA). Medical records for those patients were reviewed, and data on history of COPD, home oxygen use, smoking history, presence of DM, source of burn, inhalational injury, BMI, TBSA burned, and degree of burn was extracted. The inhalational injury was diagnosed with the presence of carbonaceous material or soot in the oropharynx with difficulty in oxygenation. The TBSA was calculated using the Wallace rule of nines [9]. The initial sample of patients obtained from IT services included all patients with a diagnosis of burn. However, patients with a misdiagnosis of burns upon chart review were excluded. These patients included those with Stevens-Johnson syndrome, road rash, frostbite, or no documentation of burns noted in the chart review. After the chart was reviewed, the final sample consisted of 1,104 patients.

Descriptive statistics were used to summarize the sample characteristics. Continuous variables were expressed as means ± standard deviations (±SD), while categorical variables were presented as numbers (N) and percentages (%). The chi-square test was utilized to determine significant differences between the two groups based on survival status for each categorical variable. For numeric variables, a Student’s t-test was applied. Logistic regression models were applied to assess the association between each predictor and survival status. Furthermore, logistic regression models are used to investigate and assess their association with the outcome of survival status. All statistical analyses were conducted using SAS (SAS 9.4, SAS Institute Inc., Cary, NC). Statistical significance was defined using a two-sided test with a p-value of <0.05.

## Results

Table 1 provides a comprehensive overview of the demographic and clinical characteristics of patients admitted to the Cabell Huntington Hospital’s BICU, stratified by their survival status. Gender distribution in the cohort, consisting of 1,104 individuals, revealed that 329 (30%) were female, with 775 (70%) being male. These gender proportions remained consistent within the subsets of survivors (N = 1,047) and deceased patients (N = 57), with no statistically significant disparities observed between the two groups. The average age for the entire cohort was 40 years (SD ± 23). Among the survivors, the mean age was 39 years (SD ± 23), while among those who did not survive, the average age notably increased to 66 years (SD ± 14) (p < 0.001). Exploring total hospital days (THD) revealed a substantial difference, with survivors averaging nine days (SD ± 15) compared to deceased patients, who averaged 13 days (SD ± 15) (p = 0.022). COPD was present in 15% of the overall sample. Among survivors, 145 (14%) had COPD, while among deceased patients, this was 14 (30%) (p < 0.001). Inhalation injuries were observed in 129 (12%) of the entire cohort, with survivors displaying a considerably lower prevalence of 102 (9.7%) compared to the 27 (47%) observed in deceased patients (p < 0.001). Significant disparities in TBSA were evident, with survivors averaging seven (SD ± 8), while deceased patients exhibited a notably higher mean TBSA of 32 (SD ± 23), signifying a substantial contrast in this measure (p < 0.001). The majority of patients (745, 67%) were diagnosed with second-degree burns. Survivors predominantly presented with second-degree burns (730, 70%), while only 15 (26%) of deceased patients displayed this type of burn. Conversely, 359 (33%) of the total sample sustained third-degree burns, with a significantly higher proportion (42, 74%) among deceased patients compared to surviving patients (p < 0.001). Various other variables, including BMI, source of burn, home oxygen, CIG (history of cigarette smoking), diabetes mellitus (DM), and additional factors, did not demonstrate statistically significant disparities between survivors and deceased patients.

**Table 1:** Sample characteristics by survival status Data presented as n (%), excluding SD. The notation “Missing N” indicates that the variable was not identified during the chart review process. This means that specific information for certain patients, such as demographics or clinical characteristics, was either not recorded or unavailable in the data collected. N, number; %, percentage; SD, standard deviation; χ2, chi-square test; THD, total hospital day; COPD, chronic obstructive pulmonary disease; CIG, history of cigarette smoking; DM, diabetes mellitus; BMI, body mass index; TBSA, total body surface area burn

Variables	Overall (N = 1,104)	Alive (N = 1,047)	Dead (N = 57)	p-value	χ2/t-score
Gender					
Female	329 (30)	310 (30)	19 (33)	0.55	0.20 (χ2 )
Male	775 (70)	737 (70)	38 (67)		
Age (mean ± SD)	40 ± 23	39 ± 23	66 ± 14	<0.001	13.78 (t-score)
THD (mean ± SD)	9 ± 15	9 ± 15	13 ± 15	0.022	1.80 (t-score)
COPD	162 (15)	145 (14)	17 (30)	<0.001	9.78 (χ2 )
Home oxygen	114 (10)	104 (9.9)	10 (18)	0.066	2.61 (χ2 )
CIG	406 (37)	387 (37)	19 (33)	0.58	0.17 (χ2 )
DM	144 (13)	132 (13)	12 (21)	0.065	2.70 (χ2 )
Inhalation injury	129 (12)	102 (9.7)	27 (47)	<0.001	70.56 (χ2 )
BMI (mean ± SD)	27 ± 8	27 ± 8	27 ± 6	0.62	-0.15 (t-score)
(Missing N)	21	19	2		
TBSA (mean ± SD)	8 ± 11	7 ± 8	32 ± 23	<0.001	8.11 (t-score)
(Missing N)	4	4	0		
Degree					
2nd degree	745 (67)	730 (70)	15 (26)	<0.001	44.46 (χ2 )
3rd degree	359 (33)	317 (30)	42 (74)		

Table 2 presents the outcomes of logistic regression models, examining the relationship between various predictor variables and survival status. Age emerged as a significant predictor, with each one-year increment associated with a 1.07 times higher risk of mortality (95% CI: 1.05, 1.09; p < 0.001). COPD patients had 2.64 times higher odds of mortality compared to those without COPD (95% CI: 1.43, 4.71; p = 0.001). Inhalation injuries were notably associated with a higher risk of mortality, with patients experiencing such injuries having 8.34 times higher odds of death (95% CI: 4.75, 14.6; p < 0.001). For each one-unit increase in TBSA, the odds of mortality increased by a factor of 1.1 (95% CI: 1.08, 1.12; p < 0.001). Patients with third-degree burns had 6.45 times higher odds of mortality compared to those with second-degree burns (95% CI: 3.60, 12.2; p < 0.001). 

**Table 2:** Association between predictors and survival status by using the logistic regression models THD, total hospital day; COPD, chronic obstructive pulmonary disease; TBSA, total body surface area burn

Variables	Odds ratio	95% CI	p-value
Age	1.07	1.05, 1.09	<0.001
THD	1.01	1.00, 1.02	0.084
COPD			
No	1	REF	REF
Yes	2.64	1.43, 4.71	0.001
Inhalation injury			
No	1	REF	REF
Yes	8.34	4.75, 14.6	<0.001
TBS	1.1	1.08, 1.12	<0.001
Degree			
2nd degree	1	REF	REF
3rd degree	6.45	3.60, 12.2	<0.001

## Discussion

Burn injuries constitute a significant public health concern globally, contributing to substantial morbidity and mortality despite advancements in medical care [1-4]. Our study underscores the persistence of this challenge, particularly within vulnerable populations such as those residing in the Appalachian region [5-7]. Through a retrospective analysis of burn patient data from Cabell Huntington Hospital’s BICU, we aimed to identify unique mortality risk factors among Appalachian burn patients and offer insights for optimizing treatment strategies and resource allocation in this underserved population.

Our findings reaffirm well-established predictors of burn mortality, including age, TBSA burned, inhalation injuries, and comorbidities such as COPD [9-11]. Consistent with previous research, advanced age was associated with increased mortality risk, highlighting the importance of age as a prognostic factor in burn care [9-11]. The presence of COPD and inhalation injuries significantly heightened mortality risk, underscoring the critical impact of pre-existing respiratory conditions and inhalational insult on burn outcomes [2].

Furthermore, our study highlights the pronounced association between the extent and severity of burns and mortality risk. Patients with larger TBSA burned and third-degree burns exhibited significantly elevated odds of mortality, reflecting the profound physiological stress and systemic complications associated with extensive burn injuries [9-10]. These findings emphasize the importance of early and accurate assessment of burn severity and prompt initiation of appropriate management strategies to mitigate adverse outcomes.

Our study also sheds light on disparities in burn outcomes between different patient subgroups. While gender distribution did not significantly influence mortality risk in our cohort, disparities based on age, comorbidities, and burn characteristics were evident. Older patients and those with pre-existing medical conditions faced heightened mortality risk, highlighting the need for tailored interventions and comprehensive care for these vulnerable populations [11].

Moreover, our study underscores the critical role of specialized burn centers, such as Cabell Huntington Hospital, in providing optimal care for burn patients in underserved regions like Appalachia. As the sole provider of burn intensive care services in the region, the hospital plays a pivotal role in managing complex burn injuries and improving outcomes for Appalachian patients.

While our study provides valuable insights into burn mortality risk factors among Appalachian patients, several limitations warrant consideration. The retrospective nature of the study limits causal inference, and the single-center design may restrict generalizability. Additionally, the study’s reliance on electronic health records introduces the potential for missing or incomplete data, impacting the accuracy and completeness of our findings.

Future research endeavors should focus on elucidating additional factors influencing burn outcomes, including socioeconomic determinants, access to care, and psychosocial factors. Collaborative efforts involving multidisciplinary teams and community stakeholders are essential for addressing the multifaceted challenges associated with burn care in underserved regions like Appalachia.

## Conclusions

In conclusion, our study highlights key mortality risk factors among Appalachian burn patients, including age, TBSA burned, inhalation injuries, COPD, and degree of burns. Tailored interventions and resource allocation are crucial to improve outcomes in underserved regions like Appalachia. Translating these findings into clinical practice, implementing targeted interventions, and fostering collaboration among healthcare providers, policymakers, and community stakeholders are essential. Future research should explore novel therapeutic strategies, socioeconomic impacts, and access to specialized care services to enhance burn care delivery and promote health equity for Appalachian burn patients and beyond.
